# Hydrodynamic cavitation coupled with zero-valent iron produces radical sulfate radicals by sulfite activation to degrade direct red 83

**DOI:** 10.1016/j.ultsonch.2023.106350

**Published:** 2023-03-02

**Authors:** Nastaran Azizollahi, Ensiyeh Taheri, Mohammad Mehdi Amin, Arvin Rahimi, Ali Fatehizadeh, Xun Sun, Sivakumar Manickam

**Affiliations:** a Department of Environmental Health Engineering, School of Health, Isfahan University of Medical Sciences, Isfahan, Iran; b Student Research Committee, School of Health, Isfahan University of Medical Sciences, Isfahan, Iran; c Environment Research Center, Research Institute for Primordial Prevention of Non-Communicable Disease, Isfahan University of Medical Sciences, Isfahan, Iran; d Key Laboratory of High Efficiency and Clean Mechanical Manufacture, Ministry of Education, School of Mechanical Engineering, Shandong University, Jinan 250061, China; e Petroleum and Chemical Engineering, Faculty of Engineering, Universiti Teknologi Brunei, Jalan Tungku Link Gadong, Bandar Seri Begawan BE1410, Brunei Darussalam

**Keywords:** Hydrodynamic cavitation, Zero-valent iron, Advanced oxidation, Direct Red 83, Decolorization, Sulfate radicals

## Abstract

In the present research, hydrodynamic cavitation (HC) and zero-valent iron (ZVI) were used to generate sulfate radicals through sulfite activation as a new source of sulfate for the efficient degradation of Direct Red 83 (DR83). A systematic analysis was carried out to examine the effects of operational parameters, including the pH of the solution, the doses of ZVI and sulfite salts, and the composition of the mixed media. Based on the results, the degradation efficiency of HC/ZVI/sulfite is highly dependent upon the pH of the solution and the dosage of both ZVI and sulfite. Degradation efficiency decreased significantly with increasing solution pH due to a lower corrosion rate for ZVI at high pH. The corrosion rate of ZVI can be accelerated by releasing Fe^2+^ ions in an acid medium, reducing the concentration of radicals generated even though ZVI is solid/originally non-soluble in water. The degradation efficiency of the HC/ZVI/sulfite process (95.54 % + 2.87%) was found to be significantly higher under optimal conditions than either of the individual processes (<6% for ZVI and sulfite and 68.21±3.41% for HC). Based on the first-order kinetic model, the HC/ZVI/sulfite process has the highest degradation constant of 0.035±0.002 min^−1^. The contribution of radicals to the degradation of DR83 by the HC/ZVI/sulfite process was 78.92%, while the contribution of *SO_4_^•−^* and *^•^OH* radicals was 51.57% and 48.43%, respectively. In the presence of HCO_3_^−^ and CO_3_^2−^ ions, DR83 degradation is retarded, whereas SO_4_^2−^ and Cl^−^ ions promote degradation. To summarise, the HC/ZVI/sulfite treatment can be viewed as an innovative and promising method of treating recalcitrant textile wastewater.

## 1 Introduction

Water pollution is one of the most critical issues that humanity faces today. Reducing the release of pollutants into water bodies requires the separation of pollutants from effluents or implementing technologies to remove them from the effluent before it is discharged into the environment [1], [2]. Textile industries are the major contributors to polluted waters containing many chemicals. Approximately 100 tonnes of textile dye wastewater are discharged worldwide annually [3]. The presence of dyes in water is visible even at a low concentration of 1 mg/L. Synthetic dye compounds are characterised by complex aromatic structures that allow them to be converted into complex compounds such as intermediates/reaction products for treatment. Therefore, dyes from industrial effluents must be removed before discharging them into the environment [4]. The most common dyes affecting human health, water bodies, soil, flora, etc., are azo dyes, characterised by one or more azo groups containing aromatic rings [1]. The azo dyes contribute 60% to total dye usage, followed by the anthraquinone dyes, classified as monoazo, disazo or trisazo, depending on the number of azo units within the structure, conjugated with aromatic or heteroaromatic rings [3]. There is evidence that direct red 83 (DR83) dye, which is an azo dye, exhibits long-lasting xenobiotic effects on ecosystems [5].

In order to remove dyes from wastewater, a variety of methods are available. Though physicochemical methods effectively decolour azo dyes, their effects on detoxifying target compounds are unknown. Therefore, a cost-effective and efficient method is a global priority [6], [7], [8]. Advanced oxidation processes (AOPs), which include ozonation, Fenton, Fenton-like, UV radiation, photocatalytic oxidation, and cavitation, are highly efficient oxidation systems for the degradation of organic compounds by generating highly oxidising free radicals such as reactive hydroxyl (*^•^OH*) and sulfate (*SO_4_^•−^*) at near-ambient temperatures and pressures [9], [10]. Using ozonation has also been found to have slower rates, higher operating costs, and limited effects on carbon content [11]. Various methods are available for treating wastewater, both as a standalone process or in conjunction with other AOPs or conventional methods, depending on the wastewater's characteristics [10]. Depending on the process type and the amount of free radicals produced, pollutant removal efficiencies can vary significantly [12].

Cavitation is a multi-bubble system with many parameters that influence the chemical and physical efficiency of the system by affecting dynamic bubbles [13]. Hydrodynamic cavitation (HC) is a green AOP technology for degrading and mineralising organic pollutants without requiring external chemicals or catalysts [14]. In the same way as acoustic cavitation, degradation ability is derived from a unique environment created by bubble collapse, which induces localised conditions with a great deal of energy release reflected in intense mechanical, thermal, and chemical phenomena [15], [16]. It has been reported by Merouani et al. [17] that the sudden collapse of microbubbles can result in high temperatures and pressures up to 5000 K and 1000 atm. It should be noted, however, that acoustic cavitation has significant limitations when degrading nonvolatile compounds [18]. Such ultrasonic devices are limited in their efficiency since a cloud of cavitation bubbles forms only near the surface of the ultrasonic source [19]. HC has been successfully used in recent years to degrade various organic pollutants in water, such as pharmaceuticals, insecticides, dyes and other phenolic compounds [20], [21]. HC’s high oxidising ability ensures no secondary load is generated during purification. It is therefore considered an energy-efficient and cost-effective technique [20]. In the HC process, cavities (bubbles) are formed, grow, and collapse on a micro-scale, releasing large amounts of energy. The efficiency of the HC process can be improved by combining it with other oxidants such as hydrogen peroxide, Fenton's reagent, and ozone [22]. Several factors influence HC efficiencies, such as velocity, pressure and device parameters [10]. Aside from its advantage in power consumption, it appears to have significant synergies in the treatment of textile wastewater when combined with other AOPs. HC combined with persulfate produced free radicals for the degradation of tetracycline synergistically [23]. A combination of HC and persulfate has also been described as a highly efficient oxidation technology for intensifying wastewater treatments [24].

Compared with other AOPs, activating sulfite salts with iron ions has many advantages; for example, it is relatively inexpensive, easy to handle, and produces harmless oxidised products [25]. It is always necessary to provide oxygen when using sulfite as a precursor to generating *SO_4_^•−^* radicals [26]. Using zero-valent iron (ZVI) as an alternative to persulfate activation has received much attention due to its ability to reduce sulfate consumption through the slow release of Fe^2+^ from Fe^0^. The combined iron-sulfite process also benefits from Fe^0^ [21]. A study investigating the potential of UV irradiation and ZVI to activate sulfite salts showed that the UV/ZVI/sulfite process effectively degraded direct red 89 dye [27].

Therefore, the main objective of this study was to evaluate the performance of a combined HC and ZVI process for the generation of sulfate radicals through sulfite activation to degrade direct red 83 (DR83) efficiently. Operational parameters, including solution pH, ZVI and sulfite dose, as well as mixed media composition, including the presence of co-existing anions such as chloride (Cl^−^), nitrate (NO_3_^−^), bicarbonate (HCO_3_^−^), carbonate (CO_3_^2−^), and sulfate (SO_4_^2−^), were evaluated to determine the degradation efficiency of DR83. Further, the quenching experiments were conducted to determine the contribution of dominant radicals to the degradation of DR83. Lastly, the rate constant of DR83 degradation by HC/ZVI/sulfite was determined using the first-order kinetic model.

## 2 Materials and methods

### 2.1 Chemicals

The commercial DR83 dye (C_33_H_20_N_6_Na_4_O_17_S_4_, *λ*_max_: 526 nm) was obtained from the Baharjin textile factory (Iran). The ZVI nanoparticles (CAS No.: 7439–89-6, particle size: 10–20 nm, and >99% purity) (Fig. S1) and sodium sulfite (Na_2_SO_3_, CAS No.: 7757–83-7, >98% purity) were obtained from Arminano (Iran) and Sigma-Aldrich (St. Louis, MO, USA), respectively. In addition, salts such as sodium chloride (NaCl, CAS No.: 7647–14-5, ≥99% purity), sodium bicarbonate (NaHCO_3_, CAS No.: 144–55-8, ≥99% purity), sodium carbonate (Na_2_CO_3_, CAS Number: 497–19-8, ≥99% purity), sodium nitrate (NaNO_3_, CAS No.: 7631–99-4, ≥99.0% purity), and sodium sulfate (Na_2_SO_4_, CAS No.: 7757–82-6, ≥99% purity) as co-existing anions were received from Merck Co. (Darmstadt, Germany). As radical scavengers, p-benzoquinone (p-BQ, C_6_H_4_(=O)_2_, CAS No.: 106–51-4, >98% purity), *tert*-butanol (TBA, (CH_3_)_3_COH, CAS No.: 75–65-0, ≥99.5% purity) and ethanol (EtOH, CH_3_CH_2_OH, CAS No.: 64–17-5, ≥95% purity) were obtained from Merck Co. (Darmstadt, Germany). Analytical grade chemicals were used without further treatment. The working solutions were prepared daily by dissolving predetermined chemicals in deionised water (DW).

### 2.2 HC system setup

In the HC setup, degradation experiments were conducted with full recirculation (Fig. S2). The HC setup consists of a feed reservoir (5 L), a centrifugal feed pump (Onyx, model: CPS 750, China), a Venturi device (length 13 cm; diameter 2.5 cm; throat diameter 0.5 mm), two pressure gauges (range 0–16 bar, Badotherm, code: 746, Netherlands), a water flowmeter (range 0.5–4 L/min, Yuyao Shunhuan Co., Ltd, China), an air compressor (rate 32 L/min, Hailea, Code: ACO-208, Guangdong Hailea Group Co. Ltd, China), and an airflow meter (range 1–10 L/min, Platon, code: GTF2BHS Roxspur Measurement & Control Ltd, England). The effluent stream was recirculated during each experiment into the feed reservoir at a constant inlet pressure and flow rate of 1.75 bar and 2 L/min, respectively.

### 2.3 Degradation experiments

In each experiment, synthetic samples were obtained by adding a specific amount of DR83 to DW. The synthetic wastewater was circulated in the HC system by the centrifugal pump. Samples were taken at regular intervals following pH adjustment with HCl or NaOH (0.1 M) and the addition of ZVI and sulfite. In order to optimise the degradation efficiency of DR83, several operational parameters (Table 1) were evaluated, including solution pH (3.0–9.0), ZVI dose (50.0–300.0 mg/L), sulfite dose (50.0–300.0 mg/L), reaction time (5–60 min), initial DR83 concentration (20.0–200.0 mg/L), and airflow (0–3.0 L/min). Following experiments, sample extracts were filtered (0.22 µm) and measured using a visible spectrophotometer (Milton Roy Company 2OD, [DR83] = 41.211 × ABS + 0.5504, *R*^2^ = 0.9997). In addition, to evaluate the effect of co-existing anions on the degradation efficiency of DR83, Cl^−^, NO_3_^−^, HCO_3_^−^, CO_3_^2−^, and SO_4_^2−^ (15.0 mM) were added to the influent solution in the HC/ZVI/sulfite process. Lastly, quenching experiments were conducted in which 15.0 mM EtOH and TBA were added to determine the relative contribution of active radicals. The experiments were conducted at ambient temperature (25±2 °C) in triplicate, and the results are presented as mean ± SD.

**Table 1:** Parametric study values for different parameters.

Parameters	Covered values	unit
pH	3.0, 4.0, 5.0, 6.0, 7.0, 8.0, 9.0	–
ZVI dose	50.0, 100.0, 150.0, 200.0, 250.0, 300.0	mg/L
Sulfite dose	50.0, 100.0, 150.0, 200.0, 250.0, 300.0	mg/L
Reaction time	5, 10, 15, 25, 35, 45, 60	min
Initial DR83 concentration	20.0, 50.0, 100.0, 150.0, 200.0	mg/L
Airflow	0, 1.5, 3	L/min

## 3 Results and discussion

### 3.1 Influence of solution pH

**Fig. 1:**
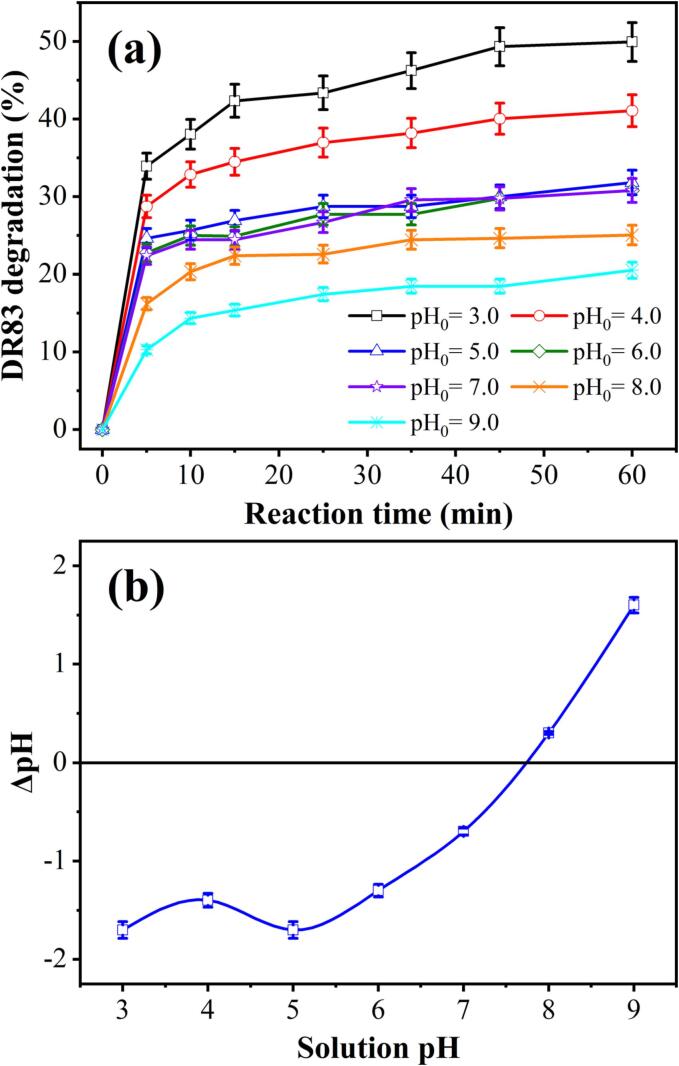
(a) Degradation of DR83 by HC/ZVI/sulfite process under different solution pH, and (b) difference in initial and final pH. Experimental conditions: [DR83]_0_ = 20.0 mg/L, [ZVI]_0_ = 50.0 mg/L, [sulfite]_0_ = 50.0 mg/L, airflow = 1.5 L/min, solution pH_0_ = 3.0–9.0, and reaction time = 5–60 min.

It is known that the solution pH is one of the determinants of the decomposition of dye molecules using ZVI corrosion by H^+^ concentration. At a constant initial DR83 concentration (20.0 mg/L), the effect of solution pH on the degradation of DR83 was investigated by varying the solution pH from 3.0 to 9.0 in the HC/ZVI/sulfite process. As shown in Fig. 1(a), increasing the pH of the solution from 3.0 to 9.0 lowered the degradation efficacy of DR83 from 49.93±2.49% to 20.55±1.03% after 60 min. Possibly, this is due to the formation of iron oxide film on the Fe^0^ surface at high pH, which in turn results in a lower release of Fe^0^. Additionally, SO_3_^2−^ ions are the dominant S(IV) species at high pH and can react slowly with iron ions, inhibiting the formation of reactive radicals. At low pH or higher concentrations of H^+^ ions, these ions react with Fe^0^, resulting in a higher concentration of Fe^2+^ in the solution [28]. In the presence of sulfite ions and a pH of 3.0, Fe^2+^ and Fe^3+^ ions are present in complex form, and Fe^2+^ ions can rapidly transform into Fe^3+^ ions. In a pH higher than 3.0, Fe^3+^ ions participate in the reaction, as shown in Eq. (1) [29]. As a result of precipitation, the dissolved iron concentration decreases, reducing degradation efficiency. In this way, Fe^3+^ tends to precipitate as the pH of the solution increases, and this polymerised and precipitated form of iron exhibits a lower reactivity than the dissolved form. Electron transfer reactions are highly pH-dependent as the main step in the oxidation of organic compounds. Anions in the deprotonated form can easily oxidise due to their relatively higher electron density [30].

(1) $$Fe^{3+}3OH^{-}\to Fe{\left(\mathrm{OH}\right)}_{3}$$

The free forms of iron (Fe^2+^ and Fe^3+^) can be generated by the reaction of Fe^0^ with water and dissolved oxygen, resulting in the production of hydrogen peroxide and *^•^OH* radicals (Eqs. (2) to (4)) [31]. The released Fe^2+^ can react with HSO_3_^−^ and SO_3_^2−^ to form FeHSO_3_^−^ (Eq. (5)), and finally, the reactive sulfate species (*SO_3_^•−^, SO_4_^•−^*, and *SO_5_^•−^*) are produced by the consecutive reactions (Eqs. (5), (6), (7), (8), (9), (10)) [27]. In addition, the reaction between *SO_4_^•−^* radicals and hydroxyl ions (OH^−^) in aqueous alkaline and acid solutions leads to *^•^OH* radical generation (Eqs. (11), (12)) [32].

(2) $$Fe^{0}+2H_{2}O\to Fe^{2+}+2OH^{-}+H_{2}$$

(3) $$Fe^{0}+O_{2}+2H^{+}\to Fe^{2+}+H_{2}$$

(4) $$Fe^{2+}+H_{2}O_{2}\to Fe^{3+}+OH^{+}+OH^{-}$$

(5) $$Fe^{2+}+HSO_{3}^{-}↔FeHSO_{3}^{+}$$

(6) $$4FeHSO_{3}^{+}+O_{2}\to FeSO_{3}^{+}+H_{2}O$$

(7) $$\mathrm{FeS}O_{3}^{+}+O_{2}\to Fe^{2+}+SO_{3}^{\cdot -}$$

(8) $$SO_{3}^{\cdot -}+O_{2}\to SO_{5}^{\cdot -}$$

(9) $$SO_{5}^{\cdot -}+HSO_{3}^{-}\to SO_{4}^{2-}+SO_{4}^{\cdot -}+H^{+}$$

(10) $$SO_{5}^{\cdot -}+SO_{3}^{\cdot -}\to SO_{4}^{\cdot -}+SO_{4}^{2-}+H^{+}$$

(11) $$SO_{4}^{∙-}\overset{\mathrm{LowpH}}{\to }+SO_{4}^{2-}+{\;}^{\cdot }OH$$

(12) $$SO_{4}^{∙-}\overset{\begin{array}{cc} \mathrm{High} & \mathrm{pH} \end{array}}{\to }+SO_{4}^{2-}+H^{+}+{\;}^{\cdot }OH$$

As displayed in Eq. (13), the combination of *^•^OH* and *SO_4_^•−^* radicals occurs under basic conditions and reduces the amount of both radicals [33]. Furthermore, at alkaline solution pH, OH^−^ with a higher amount acts as the scavenger of *^•^OH* radicals and deactivates *^•^OH* radicals (Eq. (14)) [27]. On the other hand, the redox potential of *^•^OH* radicals reduces to 2.15 V at a solution pH of 11.0, where the self-decomposition of H_2_O_2_ to H_2_O and O_2_ significantly decreases the oxidation efficiency compared to acidic conditions [34].

(13) $$SO_{4}^{\cdot -}+{\;}^{\cdot }OH\to HSO_{4}^{-}+1/2O_{2}$$

(14) $${\;}^{\cdot }OH+OH^{-}\to H_{2}O+O^{\cdot -}$$

Furthermore, at a lower pH, the dye molecules are in a molecular state with high hydrophobic properties. They reach the gas-water interface of the collapsing cavities. In the cavity interface, dye molecules are directly exposed to *^•^OH* radicals, enhancing degradation efficacy. An alkaline environment, however, causes the dye molecules to ionise and become hydrophilic, allowing them to remain in the bulk liquid [35]. Approximately 10% of the produced *^•^OH* radicals can be dispersed in the bulk solution [36], decreasing degradation efficiency. By subtracting hydrogen from protonated naphthol-OHs, dye molecules ionise, reducing the alkalinity of the solution. Since it does not accumulate at the water-cavity interface, it has less impact on the HC process [37]. In order to analyse the results, a first-order kinetic model (Eq. (15)) [38], [39] was applied, and the results are presented in Figs. S3 and S4.

(15) $$-\ln\frac{\left[DR83\right]}{{\left[DR83\right]}_{0}}=k_{\mathrm{obs}}t$$

where [DR83]_0_ and [DR83] are the initial and effluent dye concentrations at time *t* (mg/L), respectively, *k*_obs_ is the first-order rate constant (min^−1^), and *t* is the reaction time (min). As expected, the *k*_obs_ value decreases with the increasing solution pH. The order of DR83 degradation by HC/ZVI/sulfite process after 60 min is pH_0_ 3.0 (8.32±0.42×10^−3^ min^−1^) > pH_0_ 4.0 (6.03±0.29×10^−3^ min^−1^) > pH_0_ 5.0 (4.21±0.21×10^−3^ min^−1^) > pH_0_ 6.0 (4.11±0.21×10^−3^ min^−1^) > pH_0_ 7.0 (4.01±0.20×10^−3^ min^−1^) > pH_0_ 8.0 (3.31±0.14×10^−3^ min^−1^) > pH_0_ 9.0 (2.86±0.13×10^−3^ min^−1^).

Even though acidic conditions are preferred for the degradation of organic compounds by the HC/ZVI/sulfite process, considering the influence of pH on the target compounds is essential, as degradation efficiency depends on many factors, including the nature of the pollutant. Fig. 1(b) illustrates the difference between the initial and solution's pH after treatment (ΔpH) with HC/ZVI/sulfite. It could be observed that with the increase in pH from 3.0 to 9.0, ΔpH increases from −1.7 ± 0.1 to +1.6 ± 0.1. In general, the pH of the solution remains neutral. It follows that highly acidic conditions result in a higher final pH. In contrast, at an initial alkaline pH, the final pH decreases due to the formation of *^•^OH* and *H^•^* radicals and the deactivation of iron [31], [32].

### 3.2 Effect of ZVI dose

**Fig. 2:**
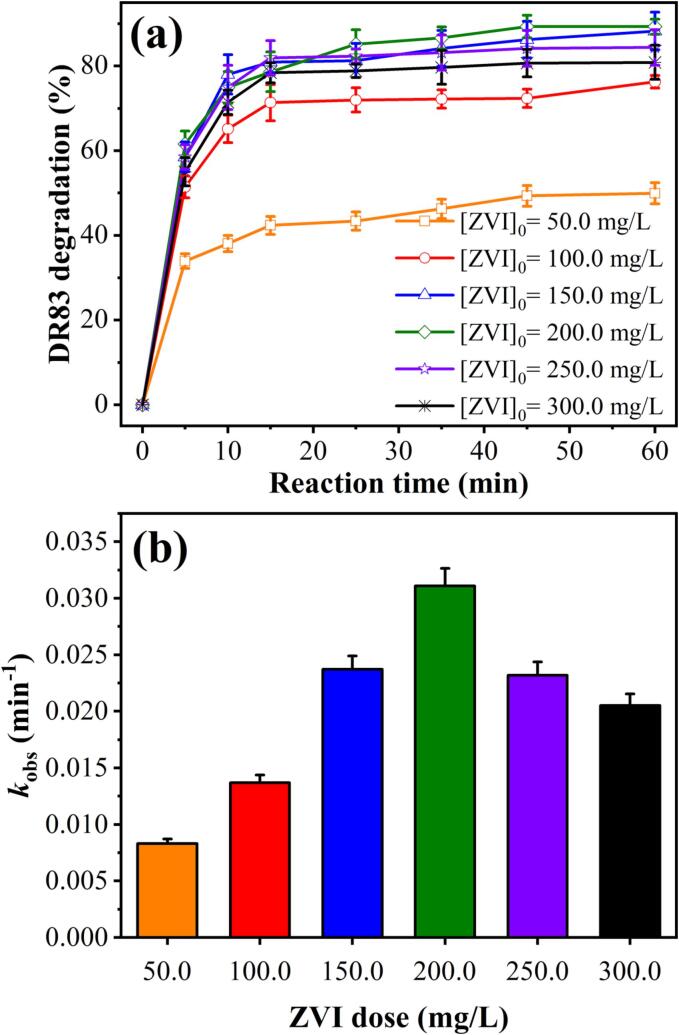
(a) Influence of ZVI dose on the degradation of DR83 through HC/ZVI/sulfite process, and (b) kinetic rate constant. Experimental conditions: [DR83]_0_ = 20.0 mg/L, [ZVI]_0_ = 50.0–300.0 mg/L, [sulfite]_0_ = 50.0 mg/L, airflow = 1.5 L/min, solution pH = 3.0, and reaction time = 50–60 min.

Using a constant initial DR83 concentration of 20.0 mg/L, a constant sulfite dose of 50.0 mg/L, and a pH of 3.0 (determined in the previous section), experiments were conducted to determine the effects of ZVI dose on degradation efficiency by changing the dose in the range of 50.0–300.0 mg/L. The results are shown in Fig. 2(a), and the kinetic study results are exhibited in Fig. 2(b) and Fig. S5. As illustrated, increasing the ZVI dose from 50.0 to 200.0 mg/L significantly improved the degradation rate from 49.93±2.49% to 89.33±1.79% after 60 min. During this period, the rate constant of DR83 degradation by the HC/ZVI/sulfite process significantly increased from 0.008±0.001 to 0.031±0.002 min^−1^. This indicates that the rate of sulfite consumption, iron release rate, and ferrous iron concentration at pH 3.0 increase with increasing ZVI loading. Therefore, the Fe^0^ addition is effective in the HC/ZVI/sulfite degradation of DR83 due to the corrosion of iron as a strong electron donor under aerobic and anaerobic conditions (Eqs. (16), (17)) [23]. Based on these equations, it can be observed that Fe^2+^ ions are generated during the corrosion of ZVI. The multivalent nature of iron enhances electron transfer, resulting in faster catalytic reactions [40]. Immediately after Fe^2+^ ions are produced, sulfite ions react in order to produce *SO_4_^•−^* radicals (Eqs. (5), (6), (7), (8), (9), (10)). Over different pH ranges, Fe^3+^ ions in several hydroxyl forms are capable of hydrolysing and forming oxide or hydroxide layers on the ZVI surface. In comparison to the homogeneous Fenton process, the recycling reaction rate (Eq. (18)) is fast [40]. Transitional iron ions, including Fe^2+^ and Fe^3+^, are always coordinated with ligands, such as H_2_O [30].

Moreover, compared with a dose of 200.0 mg/L, further increasing the ZVI dose up to 300.0 mg/L reduced the degradation of DR83 (89.33±1.79% to 80.90±4.05%) and reaction rate (0.031±0.002 to 0.021±0.001 min^−1^). This may be due to the rapid conversion of Fe^3+^ to Fe^2+^ (Eq. (18)) [40], resulting in less Fe^3+^ and more Fe^2+^. Since the high amount of Fe^2+^ ions can quench the produced *SO_4_^•−^* radicals to form sulfate ions, it causes a reduction in the overall treatment efficiency (Eq. (19)). This is in agreement with the results of Wei et al. [32], who found a low amount of Fe^2+^ but a high amount of Fe^3+^ in the solution. Additionally, as illustrated in Eqs. (2) to (4), an increase in ZVI dose can increase the production of *^•^OH* radicals [25], [41]. Compared to ZVI as a long-lasting activator, Fe^2+^ ions are not the best radical activators since they can act as a radical scavengers and have a short life since they are constantly consumed in the system. In addition, the gradual provision of reaction areas and slow release of aqueous Fe^2+^ from Fe^0^ make ZVI a good activator for the sustained oxidation of organic compounds [42]. A higher ZVI results in a lower pH as more Fe^3+^ ions are hydrolysed, resulting in more H^+^ (Eq. (20)) [32]. However, a high dose of ZVI can result in ZVI agglomeration, reducing the degradation of DR83.

(16) $$2Fe^{0}+O_{2}+2H_{2}O\to 2Fe^{2+}+4OH^{-}$$

(17) $$Fe^{0}+2H_{2}O\to Fe^{2+}+2OH^{-}+H_{2}$$

(18) $$2Fe^{3+}+Fe^{0}\to 3Fe^{2+}$$

(19) $$Fe^{2+}+SO_{4}^{\cdot -}\to Fe^{3+}+SO_{4}^{2-}$$

(20) $$Fe^{3+}+3H_{2}O\to Fe{\left(OH\right)}_{3}+3H^{+}$$

### 3.3 Effect of sulfite dose

**Fig. 3:**
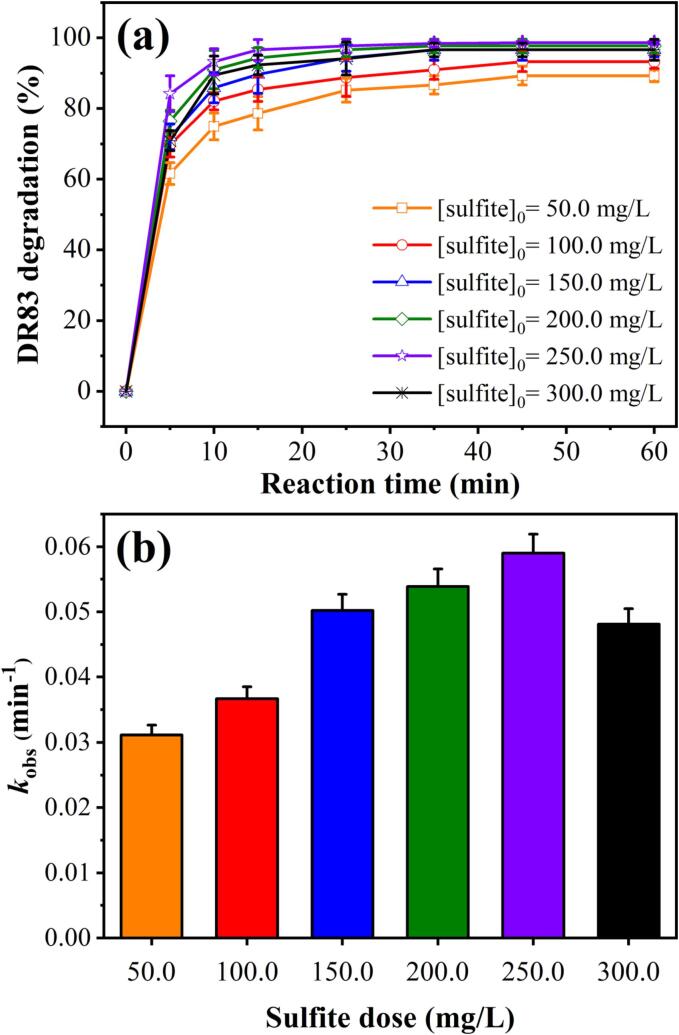
(a) Effect of sulfite dose on DR83 degradation by the HC/ZVI/sulfite process, and (b) kinetic rate constant. Experimental conditions: [DR83]_0_ = 20.0 mg/L, [ZVI]_0_ = 200.0 mg/L, [sulfite]_0_ = 50.0–300.0 mg/L, airflow = 1.5 L/min, solution pH = 3.0, and reaction time: 5–60 min.

Because *SO_4_^•−^* radicals may eliminate the target organic compounds directly or indirectly, the concentration of oxidising agent is a vital parameter for the effectiveness of AOPs [24]. Thus, in this study, the degradation efficiency of the HC/ZVI/sulfite process was evaluated as a function of sulfite dose (50.0–300.0 mg/L) at a solution pH of 3.0 with 20.0 mg/L of DR83 and 200.0 mg/L of ZVI. As shown in Fig. 3(a), a significant increase in degradation performance (from 89.33±1.79% to 98.63±0.49%) was observed by increasing the sulfite dose from 50.0 to 250.0 mg/L. Similarly, the *k*_obs_ values improved from 0.031±0.001 to 0.059±0.003 min^−1^ (Fig. 3(b) and Fig. S6). The observed trend is presumably related to the formation of FeHSO_3_^+^ (Eq. (5)) and the highly sulfite-dose-dependent reaction rate between SO_5_^2−^ and HSO_3_^−^ [43]. The increase in sulfite ions dose is associated with the improved reaction between iron and sulfite, which enhances the formation of FeSO_3_^+^ (Eq. (21)) and also *^•^OH*, *SO_4_^•−^*, and *SO_5_^•−^* radicals (Eqs. (7), (8), (9), (10), (11), (12)) [28]. In the system, the generated sulfate radicals can react with more water molecules to produce more [44].

However, when the sulfite dose was increased from 250.0 mg/L to 300.0 mg/L, DR83 degradation slightly decreased from 98.63±0.49% to 96.61±2.90%. This inhibition may be ascribed to the quenching of *SO_4_^•−^* and *^•^OH* radicals with HSO_3_^−^/SO_3_^2−^ (Eqs. (22), (23)) [31]. Although SO_3_^2−^ ions are more reactive than sulphnate ions (HSO_3_^−^), sulphnate ions are more stable in gaseous and aqueous environments [30]. Furthermore, sulfite ions are required in the HC/ZVI/sulfite process for the generation of *SO_4_^•−^* and *^•^OH* radicals, while a dose higher than the optimal value may result in sulfite participation, resulting in lower removal efficiency [28]. Sulfite ions can react with Fe ions to form a Fe complex by bonding to the metal ions by one single atom (S or O) or two atoms (O and O, or S and O). The lower electronegativity of S atoms makes S-bonded complexes more stable than O-bonded complexes. Alternatively, Fe can form a complex with S via Fe-S linkage. Fe-sulphite complexes are significantly more stable than Fe-sulphate complexes [30]. As a result of the above factors, degradation performance is reduced. Chen et al. [45] also observed a similar trend, noting that the concentration of Na_2_SO_3_ increased in response to a reaction between *SO_4_^•−^* and HSO_5_^•−^ with Na_2_SO_3_, which reduced the decolourisation efficiency.

(21) $$Fe^{3+}+HSO_{3}^{-}\to FeSO_{3}^{+}+H^{+}$$

(22) $$SO_{4}^{\cdot -}+SO_{3}^{2-}\to SO_{3}^{\cdot -}+OH^{-}$$

(23) $${\;}^{\cdot }OH+SO_{3}^{2-}\to SO_{3}^{\cdot -}+OH^{-}$$

### 3.4 Effect of initial concentration of DR83

**Fig. 4:**
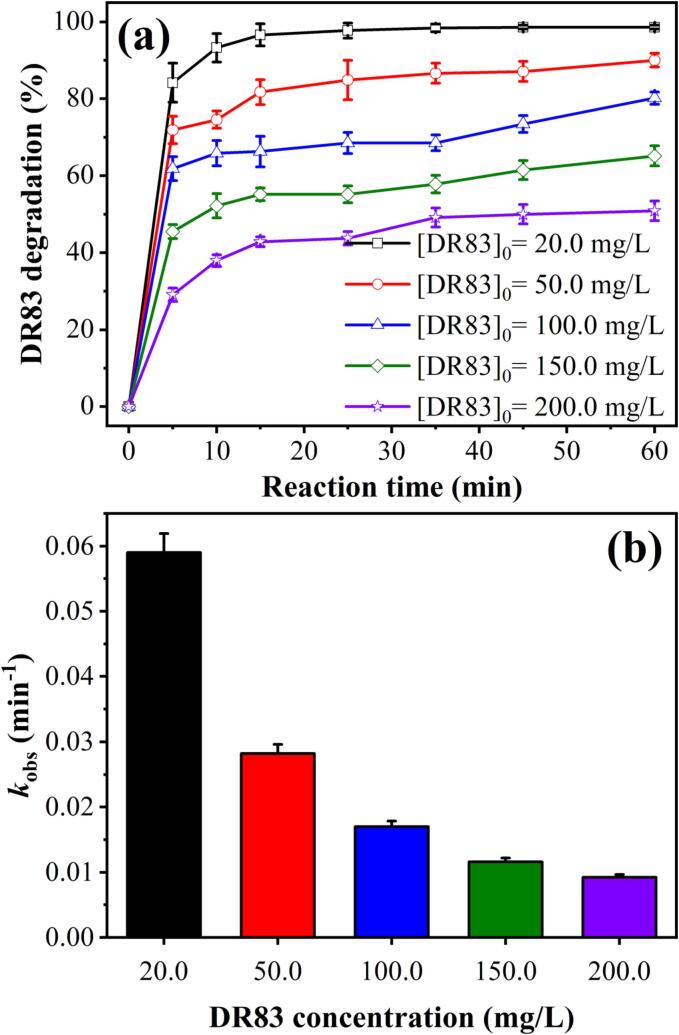
(a) Influence of the initial DR83 concentration on DR83 degradation through the HC/ZVI/sulfite process and (b) kinetic rate constant. Experimental conditions: [DR83]_0_ = 20.0–200.0 mg/L, [ZVI]_0_ = 200.0 mg/L, [sulfite]_0_ = 250.0 mg/L, airflow = 1.5 L/min, solution pH = 3.0, and reaction time = 5–60 min.

The degradation performance of the HC/ZVI/sulfite process has been evaluated for various wastewaters' varying strengths. Under the optimised conditions (pH = 3.0, ZVI dose = 200.0 mg/L, and sulfite dose = 250.0 mg/L), the effect of the initial concentration of DR83 from 20.0 to 200.0 mg/L on the degradation efficiency was assessed (Fig. 4(a)). Degradation efficiency is directly proportional to the initial concentration of DR83. As the concentration increased from 20.0 to 200.0 mg/L, the degradation efficiency reduced from 98.63±0.49% to 50.89±2.54%. Nevertheless, the amount of DR83 eliminated increased with increasing initial dye concentration. For example, after 60 min of reaction, the removal efficiency was 98.63±0.49%, and 19.73 mg/L of DR83 molecules (20.0 mg/L) were thus removed. In addition to the decrease in removal efficiency, the amount of DR83 eliminated increased from 45.01 to 80.18, 97.73 to 101.78 mg/L as the initial dye concentration was increased to 50.0, 100.0, 150.0 and 200.0 mg/L. Similar reports have been on the degradation of naphthol blue black by sonolysis [44]. An increase in the total amount of DR83 may cause it. In contrast, the relative amount of produced radicals is constant as the operating conditions, such as ZVI, sulfite, and initial pH, are identical. In addition, the reaction possibility between DR83 molecules and *SO_4_^•−^* and *^•^OH* radicals would decrease with increasing DR83 concentration. Since the higher dye concentration consumes more active radicals, the amount of total radicals and hence the degradation efficiency of the HC/ZVI/sulfite process decreases. Likewise, *k*_obs_ decreased from 0.059±0.003 to 0.0092±0.0004 min^−1^, with the increasing DR83 concentration from 20.0 to 200.0 mg/L (Fig. 4(b) and Fig. S7). The observed steepness trend in the degradation efficiency with increasing dye concentration is consistent with an earlier study [19].

### 3.5 Effect of air purging

**Fig. 5:**
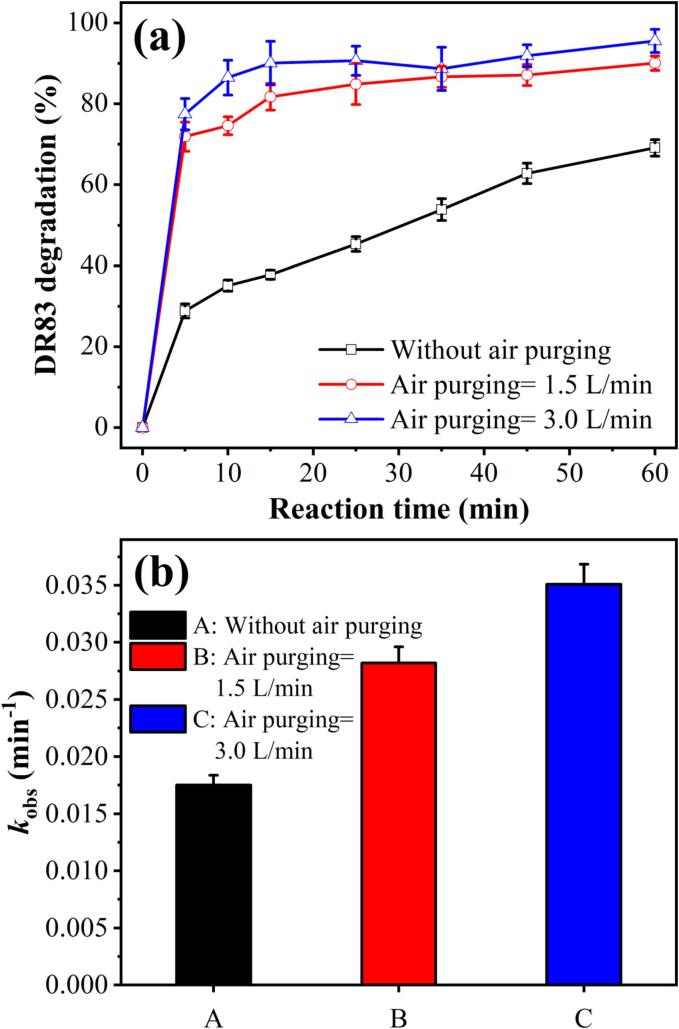
(a) Influence of air purging on DR83 degradation using HC/ZVI/sulfite process and (b) kinetic rate constant. Experimental conditions: [DR83]_0_ = 50.0 mg/L, [ZVI]_0_ = 200.0 mg/L, [sulfite]_0_ = 250.0 mg/L, solution pH = 3.0, and reaction time = 5–60 min.

Airflow was evaluated as one of the influencing factors that affect DR83 degradation at a concentration of 50.0 mg/L of DR83 and a pH of 3.0, and the results are shown in Fig. 5(a). Approximately 90.03±1.81% and 95.54±2.87% DR83 were degraded with an air purging rate of 1.5 and 3.0 L/min, respectively, compared to 69.1±2.07% without air purging. As oxygen reacts with water molecules, it creates *^•^OH* radicals, which promote the treatment process [46]. The oxygen (O_2_) molecules act as the ultimate electron acceptors and can react with *SO_3_^•−^* to generate *SO_5_^•−^*, which is the precursor of *SO_4_^•−^* radicals (Eqs. (8), (9), (10)) [28]. Therefore, dissolved oxygen (DO) is a significant influencing factor and indirectly affects the decolourisation of DR83. Compared to without air purging, the air purging of the solution led to an increased degradation reaction rate (Fig. 5(b) and Fig. S8). Moreover, air provides additional nuclei that increase the cavitational intensity and cause further degradation [47]. There have been similar findings in the presence of O_2_ and Fe^3+^ where S_2_O_6_^2−^ generation increased as an intermediate product [30]. Thus, the increasing air purging enhanced the efficiency of DR83 degradation, and the air purging at the rate of 3.0 L/min was selected for the remaining experiments.

### 3.6 Kinetics of DR83 degradation under optimal conditions by individual and combined processes

**Fig. 6:**
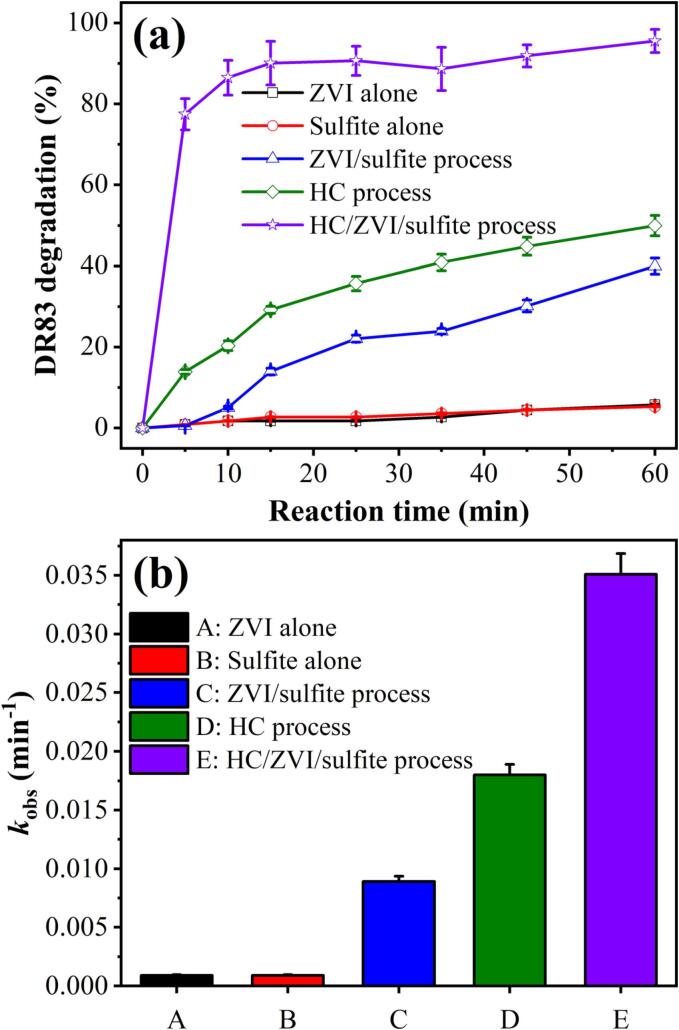
(a) The degradation of DR83 using sulfite, ZVI, HC, ZVI/sulfite, and HC/ZVI/sulfite processes and (b) kinetic rate constant. Experimental conditions: [DR83]_0_ = 50.0 mg/L, [ZVI]_0_ = 200.0 mg/L, [sulfite]_0_ = 250.0 mg/L, pH = 3.0, airflow = 3.0 L/min, and reaction time = 5–60 min.

For determining the impact of individual and combined processes, the degradation experiments were performed with ZVI and sulfite alone, HC, ZVI/sulfite, and HC/ZVI/sulfite processes at a DR83 concentration of 50.0 mg/L and a solution pH of 3.0. As shown in Fig. 6(a), no obvious degradation of DR83 was observed after 60 min of individual ZVI and sulfite treatments (<6%), which is consistent with previous studies [26], [28]. It is due to the absence of sulfite activation that generates active radicals. The ZVI/sulfite process can degrade DR83 under optimal conditions ([ZVI]_0_ = 200.0 mg/L and [sulfite]_0_ = 250.0 mg/L). The degradation of DR83 by 40.01±2.01% using the ZVI/sulfite process might be attributed to the activation of sulfite salts which generate *SO_3_^•−^* radicals that are the precursor of *SO_4_^•−^* radicals (Eqs. (8), (9), (10)) [26]. With the application of the HC process, the degradation efficiency of DR83 was significantly improved to 49.94±2.49% due to the decomposition of DR83 molecules by the produced active radicals, mainly that of *^•^OH* radicals (Eq. (24)) [24]. According to the previous study, HC degrades target contaminants by two main mechanisms. Firstly, there is pyrolysis in a cavitation bubble where high pressure and temperature are generated. The second is the oxidation of *^•^OH* radicals at the bubble interface at a lower temperature than the bubble interior. Sonochemical reactions can be initiated under the resultant conditions [24], [48].

However, when ZVI and sulfite ions were added to the HC process, the degradation efficiency of DR83 significantly increased from 68.21±3.41% to 95.54±2.87%. It is due to the enhancement of mass transfer by the HC process and the acceleration of the oxidation process by increasing the exposure of ZVI [23]. Furthermore, the HC/ZVI/sulfite process can promote the hydrolysis reaction of sulfite in an acidic environment, generating more Fe^2+^ ions [49].

(24) $$H_{2}O\overset{\mathrm{HCprocess}}{\to }{\;}^{\cdot }OH+H^{\cdot }$$

As shown in Fig. 6(b) and Fig. S9, DR83 degradation through different processes was analysed using first-order kinetics (Eq. (15)). The *k*_obs_ values for DR83 degradation by ZVI, sulfite, HC, and HC/ZVI/sulfite processes are 0.0009±0.00001, 0.0009±0.00001, 0.018±0.001, and 0.035±0.002 min^−1^, respectively. Based on Eq. (25), a comparison of rate constants of first-order kinetics of ZVI, sulfite, and HC processes during DR83 degradation was carried out to better understand the synergistic effect (*SF*) between these processes.

(25) $$\mathrm{SF}=\frac{k_{\mathrm{com}.p}}{k_{\mathrm{ind}.p(1)}+k_{\mathrm{ind}.p(2)}+k_{\mathrm{ind}.p(n)}}$$

where *k_com.p_* and *k_ind.p_* are the rate constants for the HC/ZVI/sulfite and individual processes, respectively. It was observed that ZVI, sulfite, and HC processes led to a synergistic kinetic effect in DR83 degradation by the HC/ZVI/sulfite process, with an SF value of 1.77.

The amount of total organic carbon (TOC) in the initial solution and effluent was measured to verify the mineralisation of DR83 by the HC/ZVI/sulfite process. The results of the experiment demonstrate the reduction in TOC of 69.21±3.01% by the HC/ZVI/sulfite process after 60 min under optimal conditions ([ZVI]_0_ = 200.0 mg/L, [sulfite]_0_ = 250.0 mg/L, pH = 3.0, and airflow = 3.0 L/min). In the presence of ZVI and HC, sulfite salts were activated to generate free radicals, destroying the C-X bond in the DR83 molecule and its intermediates. Additionally, the possibility of recovering ZVI from the HC/ZVI/sulfite process was investigated (data are not shown). Based on the results, a relatively good recovery of ZVI was achieved. Table 2 summarises the comparison of DR83 degradation by the HC/ZVI/sulfite process and other AOPs. Compared with other AOPs, the HC/ZVI/sulfite process achieved a higher rate of DR83 degradation with lower chemical consumption. As a result, the developed HC/ZVI/sulfite process is promising for efficiently treating recalcitrant textile wastewater.

**Table 2:** A summary of the removal of azo dyes by different AOPs.

Process	Experimental conditions	Performance		Reference
		Removal (%)	*k*_obs_ (min^−1^)	
HC/ZVI/sulfite	[DR83]_0_ = 50.0 mg/L, [ZVI]_0_ = 200.0 mg/L, [sulfite]_0_ = 250.0 mg/L, pH = 3.0, airflow = 3.0 L/min, and reaction time = 60 min	95	0.035	Present study
UV/Cl	[DR83]_0_ = 50.0 mg/L, [Cl]_0_ = 1000.0 mM, pH = 3.0, UV fluence = 6.0 mW/cm^2^, and reaction time = 60 min	93	0.025	[7]
PL/H_2_O_2_	[DR83]_0_ = 30.0 mg/L, [H_2_O_2_]_0_ = 900.0 mg/L, fluence = 96. J/cm^2^, and pH = 11.0	76	0.027	[1]
Electrooxidation	[DR83]_0_ = 150.0 mM, NaCl = 5.0 N, density = 5.0 mA/cm^2^, and contact time = 60 min	–	0.074	[4]
US/PS	[Naphthol Blue Black]_0_ = 5 mg/L, [PS]_0_ = 0.5–1.0 mg/L, frequency = 585 kHz, power = 80 W, and reaction time = 20 min	95	–	[50]
HC/ZVI/H_2_O_2_	[Orange G] _0_ = 40 mg/L, [ZVI]_0_ = 0.7 g/L, [H_2_O_2_]_0_ = 0.01 g/L, pH = 3.0, and reaction time = 20 min	74.6	–	[51]
HC/Fenton	FeSO_4_·7H_2_O:H_2_O_2 =_ 1:5, pH = 6.8, pressure = 5 bar and reaction time = 60 min	97.7	0.002	[52]

### 3.7 Identification of radicals and the effect of the solution matrix

**Fig. 7:**
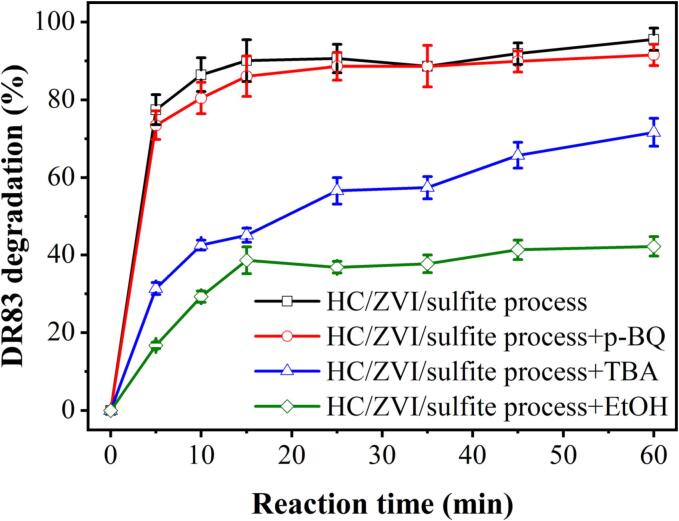
Effect of radical scavengers on DR83 degradation by the HC/ZVI/sulfite system. Experimental conditions: [DR83]_0_ = 50.0 mg/L, [ZVI]_0_ = 200.0 mg/L, [sulfite]_0_ = 250.0 mg/L, pH = 3.0, airflow = 3.0 L/min, concentration of scavengers = 15 mM and reaction time = 5–60 min.

Radical scavengers usually possess electron-rich structures that enable them to easily react with radicals such as C π bonds, aromatic rings, and hydroxyl groups. Radicals can be identified by fast-trapping specific radical groups [30]. In this study, the types of active free radicals generated by the HC/ZVI/sulfite process and their contribution to the degradation of DR83 using the radical scavengers p-BQ (superoxide radical (*O_2_^•−^*) scavenger), EtOH and TBA, which are primarily the *SO_4_^•−^* and *^•^OH* radicals, were determined. It is well known that TBA is an effective scavenger of *^•^OH* radicals, and EtOH is effective in quenching both *SO_4_^•−^* and *^•^OH* radicals [53]. In Fig. 7, it can be seen that the addition of p-BQ to the HC/ZVI/sulfite reaction medium did not significantly suppress the DR83 degradation of CIP. After 60 min, the degradation of DR83 decreased from 95.54±2.87% to 91.54±2.75% in the presence of 15 mM p-BQ. Unlike the control (95.54±2.87%), DR83 degradation is notably inhibited by 15.0 mM EtOH and TBA, decreasing to 42.26±2.54% and 71.63±3.58%. It illustrates the dominant role played by *SO_4_^•−^* and *^•^OH* radicals. Using Eqs. (26), (27), (28), (29) from the study of Fadaei et al. [54], the partial contributions of *SO_4_^•−^* and *^•^OH* radicals were estimated based on the rate constant of first-order kinetics (Table 3 and Fig. S10). As determined by calculations, radicals contributed 78.92% to the degradation of DR83 during the HC/ZVI/sulfite process; the contributions of *SO_4_^•−^* and *^•^OH* radicals were 51.57% and 48.43%, respectively. It must be noted that the scavengers may trap various radicals simultaneously. There was no consistency in the concentration of scavengers found in different studies because their effects depend on their concentration. Quenching experiments can be used to investigate the role of different radicals in the HC/ZVI/sulfite systems, which is simple and inexpensive [55].

(26) $$\mathrm{Radical}\;contribution\;\left(\%\right)=\frac{k_{\mathrm{obs}}-k_{\mathrm{obs}+EtOH}}{k_{\mathrm{obs}}}\times 100$$

(27) $$\mathrm{HC}\;effect\;\left(\%\right)=100-Radical\;contribution\;\left(\%\right)$$

(28) $${\;}^{\cdot }OH\;contribution\;\left(\%\right)=\frac{k_{\mathrm{obs}}-k_{\mathrm{obs}+TBA}}{k_{\mathrm{obs}}}\times 100$$

(29) $$\begin{array}{c} SO_{4}^{\cdot -}contribution\;\left(\%\right)=Radical\;contribution\;\left(\%\right)\\ {-}^{\cdot }OH\;contribution\;\left(\%\right) \end{array}$$

**Table 3:** Kinetic rate of DR83 degradation by the HC/ZVI/sulfite process in the presence of radical scavengers.

Item	Value
*k*_obs_ value of HC/ZVI/sulfite process	0.035±0.002 (min^−1^)
*k*_obs_ value of HC/ZVI/sulfite process + p-BQ	0.030±0.002 (min^−1^)
*k*_obs_ value of HC/ZVI/sulfite process + TBA	0.018±0.001 (min^−1^)
*k*_obs_ value of HC/ZVI/sulfite process + EtOH	0.007±0.0004 (min^−1^)
*k*_obs_ value of HC process	0.018±0.001 (min^−1^)
Radical contribution	78.92 (%)
HC process	21.08 (%)
Contribution of *^•^OH* radicals	48.43 (%)
Contribution of *SO_4_^•−^* radicals	51.57 (%)

**Fig. 8:**
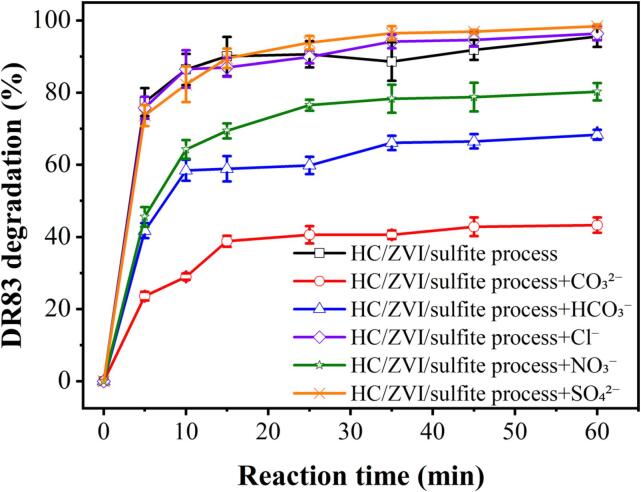
Effect of co-existing anion on DR83 degradation by the HC/ZVI/sulfite process. Experimental conditions: [DR83]_0_ = 50.0 mg/L, [ZVI]_0_ = 200.0 mg/L, [sulfite]_0_ = 250.0 mg/L, pH = 3.0, airflow = 3.0 L/min, concentration of anions = 15 mM and reaction time = 5–60 min.

Natural water resources typically contain anions such as Cl^−^, NO_3_^−^, HCO_3_^−^, CO_3_^2−^, and SO_4_^2−^. These anions can affect the degradation performance of AOPs. As shown in Fig. 8, co-existing anions impact the degradation of DR83 in the HC/ZVI/sulfite process. Table 4 (Fig. S11) summarises the first-order kinetics and corresponding rate constants. Cl*^−^* and SO_4_^2^*^−^* ions generally promoted DR83 degradation, while NO_3_^−^, HCO_3_^−^, and CO_3_^2−^ inhibited it. After adding HCO_3_^−^ and CO_3_^2−^ ions to the feed solution, after 60 min, the degradation efficiency of DR83 significantly declined from 95.54±2.87% to 68.34±1.37% and from 95.54±2.87% to 43.28±2.16%, respectively. This is presumably related to the scavenging nature of *HCO_3_*^–^ and *CO_3_^2^*^–^ ions for both *SO_4_^•−^* and *^•^OH* radicals, leading to the production of *CO_3_^•–^* radicals with lower redox potential (*E*^0^ = 1.78 V) (Eqs. (30), (31), (32), (33)) [56]. Furthermore, the hydrolysis of *HCO_3_*^–^ and *CO_3_^2^*^–^ ions at 15.0 mM may change the solution pH from acidic to alkaline. This result inhibits the generation of *SO_4_^•−^* and *^•^OH* radicals. Instead, it produces carbonate radicals [57]. Therefore, the degradation effects of DR83 are not satisfactory.

(30) $$SO_{4}^{\cdot -}+HCO_{3}^{-}\to SO_{4}^{2-}+CO_{3}^{\cdot -}+H^{+}$$

(31) $${\;}^{\cdot }OH+HCO_{3}^{-}\to CO_{3}^{\cdot -}+H_{2}O$$

(32) $$CO_{3}^{2-}+{\;}^{\cdot }OH\to CO_{3}^{\cdot -}+OH^{-}$$

(33) $$SO_{4}^{\cdot -}+CO_{3}^{2-}\to SO_{4}^{2-}+CO_{3}^{\cdot -}$$

**Table 4:** Kinetic rate of DR83 degradation by the HC/ZVI/sulfite process in the presence of co-existing anions.

Process type	*k*_obs_ (min^−1^)	Synergistic effect
HC/ZVI/sulfite process (control)	0.035±0.002	–
HC/ZVI/sulfite process with CO_3_^2−^ addition	0.007±0.001	0.21
HC/ZVI/sulfite process with HCO_3_^−^ addition	0.014±0.001	0.41
HC/ZVI/sulfite process with NO_3_^−^ addition	0.023±0.001	0.64
HC/ZVI/sulfite process with Cl^−^ addition	0.044±0.002	1.26
HC/ZVI/sulfite process with SO_4_^2−^ addition	0.055±0.003	1.57

In the HC/ZVI/sulfite process, due to adding NO_3_^−^ ions, the degradation efficiency of DR83 decreased from 95.54±2.87% to 80.33±2.41%. In the feed solution, NO_3_^−^ ions can be converted to nitrate radicals when they interact with active species such as *SO_4_^•−^* and *^•^OH* radicals (Eqs. (34), (35), (36), (37), (38)), as well as quenching of *^•^OH* radicals (Eq. (39)) [34], [56].

(34) $$NO_{3}^{-}\overset{\mathrm{HC}}{\to }ONOO^{-}$$

(35) $$\mathrm{ONO}O^{-}+{\;}^{\cdot }OH\to NO^{\cdot }+O_{2}+HO^{-}$$

(36) $$NO_{3}^{-}\overset{\mathrm{HC}}{\to }NO_{2}^{\cdot }+O^{\cdot -}$$

(37) $$NO_{3}^{-}+{\;}^{\cdot }OH\to NO_{3}^{\cdot -}+OH^{-}$$

(38) $$NO_{3}^{-}+SO_{4}^{\cdot -}\to NO_{3}^{\cdot -}+SO_{4}^{2-}$$

(39) $$NO^{\cdot }+{\;}^{\cdot }OH\to HO_{2}NO$$

Adding Cl^−^ ions (as halide) increased the degradation efficiency of DR83 from 95.54±2.87% to 96.44±1.93%. As a result of adding Cl^−^ ions, the reaction between *^•^OH* radicals and Cl^−^ ions resulted in the formation of *Cl^•^* radicals (*E*^0^ = 2.4 V), which were then converted into *Cl_2_^•^*^−^ radicals (*E*^0^ = 2.0 V) through the reaction with *HClO* (Eqs. (40), (41), (42), (43)) [54], [58], improving the degradation efficiency of target contaminants. Further, adding Cl^−^ ions would facilitate the corrosion of Fe^0^ to Fe^2+^ by increasing the ion strength [26].

(40) $$Cl^{-}+{\;}^{\cdot }OH\to HClO^{\cdot -}$$

(41) $$\mathrm{HCl}O^{\cdot -}+H^{+}\to HClOH^{\cdot }$$

(42) $$\mathrm{HClO}H^{\cdot }\to Cl^{\cdot }+H_{2}O$$

(43) $$Cl^{\cdot }+Cl^{-}\to Cl_{2}^{\cdot -}$$

In the presence of 15.0 mM SO_4_^2−^ ions, the degradation efficiency of DR83 increased from 95.54±2.87% to 98.43±0.49% after 60 min. The reason for this is the presence of surplus *SO_4_^•−^* radicals formed by the reaction between SO_4_^2−^ ions and *^•^OH* radicals (Eq. (44)) [59], [60], which could degrade more DR83 molecules, thereby improving degradation efficiency slightly.

(44) $${\;}^{\cdot }OH+SO_{4}^{2-}\to OH^{-}+SO_{4}^{\cdot -}$$

### 3.8 Variation of the absorption spectrum of DR83 dye

**Fig. 9:**
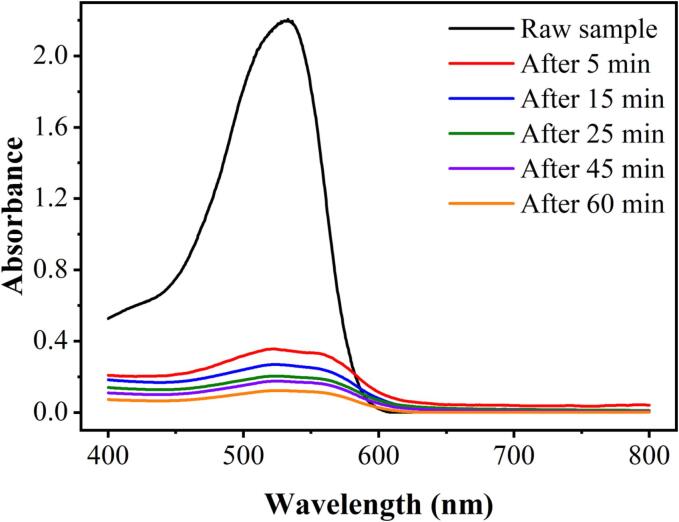
Variation of the visible absorbance spectrum of DR83 dye during the HC/ZVI/sulfite process. Experimental conditions: [DR83]_0_ = 50.0 mg/L, [ZVI]_0_ = 200.0 mg/L, [sulfite]_0_ = 250.0 mg/L, pH = 3.0, air flow = 3.0 L/min, and reaction time = 5–60 min.

Fig. 9 shows the absorption curves of DR83 under optimal operating conditions during the HC/ZVI/sulfite treatment under different reaction times. Compared with the raw sample, the intensity of the bands in the visible spectrum of DR83 dye significantly decreased after 5 min. According to the spectrum, DR83 exhibits an absorbance peak at 540 nm, attributed to the azo linkage in the chromophore group. In addition, a significant decrease in absorbance was observed after 5 min, indicating the degradation of DR83 dye’s azo structure and aromatic molecules, as demonstrated by earlier studies [1], [7].

## 4 Conclusions

This study aimed to determine the effectiveness of the HC/ZVI/sulfite process for the degradation of DR83. As a result of the HC alone, over half of the DR83 (68.21±3.41%) was degraded. The effectiveness of this method improved to 95.54±0.87 based on ZVI and sulfite at an initial concentration of 50 mg/L. A significant improvement in the degradation of DR83 from 49.93±2.49% to 89.33±1.79% was observed when the ZVI dose was increased from 50.0 mg/L to 200.0 mg/L after 60 min, which reduced when the ZVI dose was increased to 300.0 mg/L (decreased by 10%). Similarly, the degradation performance of DR83 was enhanced from 89.33±1.79% to 98.63±0.49% by increasing the sulfite dose from 50.0 to 250.0 mg/L. Despite this, the degradation efficiency was reduced (by 3%) by the quenching effect of surplus HSO_3_^−^/SO_3_^2−^. The presence of NO_3_^−^, HCO_3_^−^, and CO_3_^2−^ ions in the solution inhibited DR83 degradation. However, the SO_4_^2−^ and Cl^−^ ions in the solution enhanced degradation efficiency. Therefore, the synergistic effect of the HC/ZVI/sulfite process relates to generating powerful oxidants, including *SO_4_^•−^* and *^•^OH* radicals. The DR83 dye, however, did not display an absorbance peak at 540 nm, indicating that the azo structure and aromatic molecules have degraded.

## Declaration of Competing Interest

The authors declare that they have no known competing financial interests or personal relationships that could have appeared to influence the work reported in this paper.

## Data Availability

Data will be made available on request.
