# Supplementary material for: Hydrodynamic cavitation coupled with zero-valent iron produces radical sulfate radicals by sulfite activation to degrade direct red 83
Source: Ultrason Sonochem. 2023 Mar 2;95:106350. doi: 10.1016/j.ultsonch.2023.106350 (PMC10014301; doi:10.1016/j.ultsonch.2023.106350)
Supplement: Supplementary data 1 [file mmc1.doc]

**Supplementary Material**

**Hydrodynamic cavitation coupled zero-valent iron for the production of sulfate radicals through sulfite activation for the degradation of Direct Red 83**

Nastaran Azizollahi^a,b^, Ensiyeh Taheri^a,c^, Mohammad Mehdi Amin^a,c^, Arvin Rahimi^a,b^,

Ali Fatehizadeh^a,c,*^, Xun Sun^d,*^, Sivakumar Manickam^e,*^

^a^Department of Environmental Health Engineering, School of Health, Isfahan University of Medical Sciences, Isfahan, Iran,

^b^Student Research Committee, School of Health, Isfahan University of Medical Sciences, Isfahan, Iran

^c^Environment Research Center, Research Institute for Primordial Prevention of Non-Communicable Disease, Isfahan University of Medical Sciences, Isfahan, Iran

^d^Key Laboratory of High Efficiency and Clean Mechanical Manufacture, Ministry of Education, School of Mechanical Engineering, Shandong University, Jinan 250061, China

^e^Petroleum and Chemical Engineering, Faculty of Engineering, Universiti Teknologi Brunei, Jalan Tungku Link Gadong, Bandar Seri Begawan, BE1410, Brunei Darussalam

* Corresponding authors.

E-mail addresses: [a.fatehizadeh@hlth.mui.ac.ir](mailto:a.fatehizadeh@hlth.mui.ac.ir) (A. Fatehizadeh), [xunsun@sdu.edu.cn](mailto:xunsun@sdu.edu.cn) (X. Sun), [manickam.sivakumar@utb.edu.bn](mailto:manickam.sivakumar@utb.edu.bn) (S. Manickam).

**LIST OF FIGURES**

**Fig. S1.** Characteristics of ZVI; (a) and (b) SEM image and (c) EDS spectrum

**Fig. S2.** Schematic diagram of the HC system.

**Fig. S3.** First-order kinetic curves of the degradation of DR83 under different solution pH.

**Fig. S4.** Effect of solution pH on the kinetic rates of DR83 degradation.

**Fig. S5.** First-order kinetic curves of the degradation of DR83 under different doses of ZVI.

**Fig. S6.** First-order kinetic curves of the degradation of DR83 under different sulfite doses.

**Fig. S7.** First-order kinetic curves of the degradation of DR83 under different concentrations of DR83.

**Fig. S8.** First-order kinetic curves of the degradation of DR83 under different air purging.

**Fig. S9.** First-order kinetic curves of the degradation of DR83 through different processes.

**Fig. S10.** First-order kinetic curves of the degradation of DR83 in the presence of radical scavengers.

**Fig. S11.** Influence of coexisting anions on DR83 degradation.

| 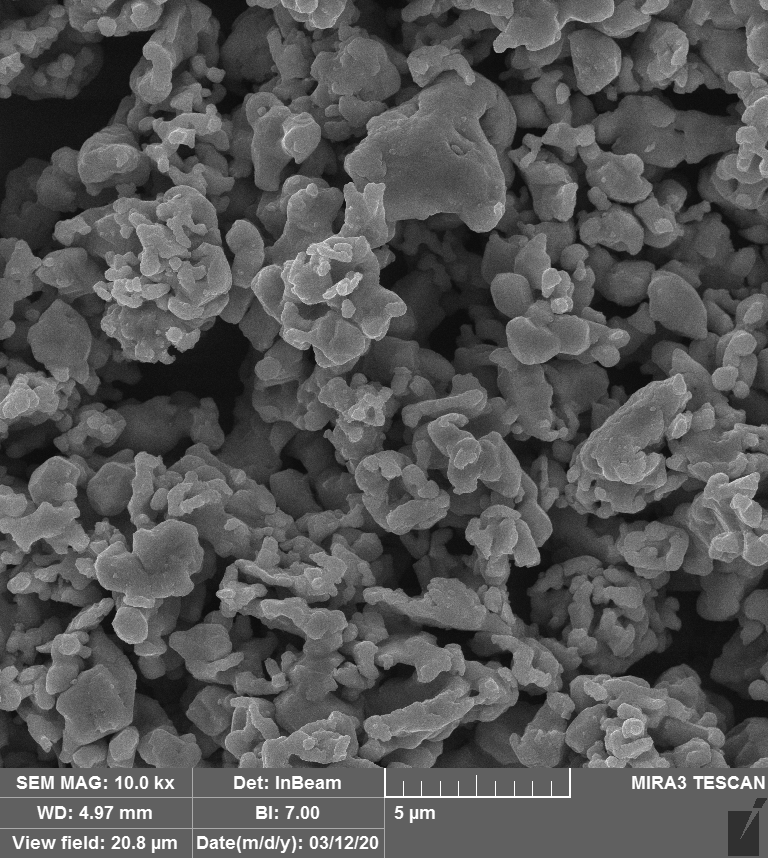  **(a)** | 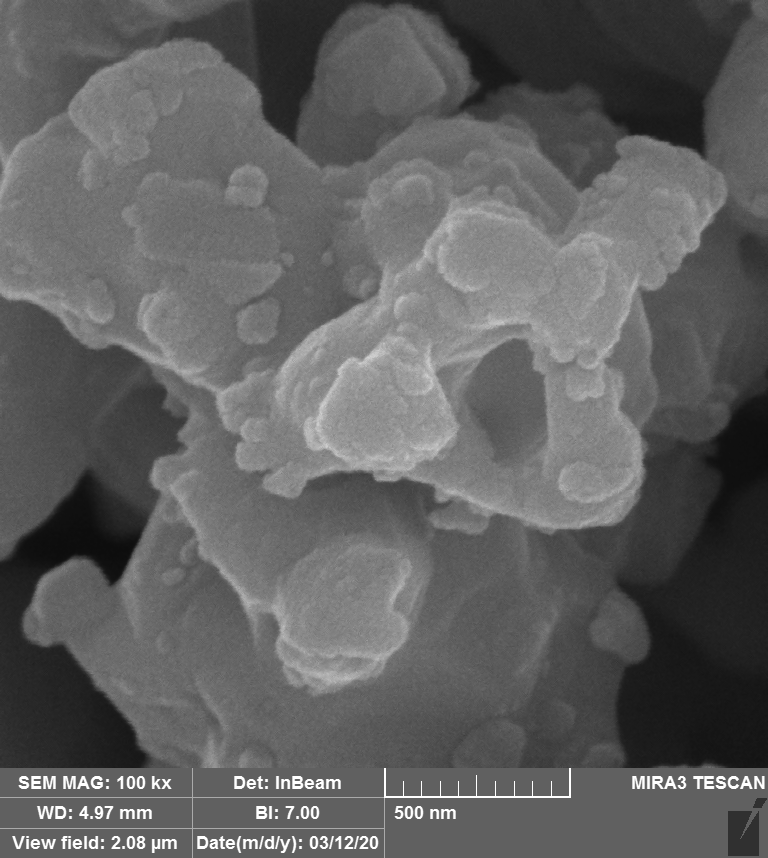  **(b)** |
| --- | --- |
| **(c)** | |

Fig. S1. Characteristics of ZVI: (a) and (b) SEM image and (c) EDS spectrum


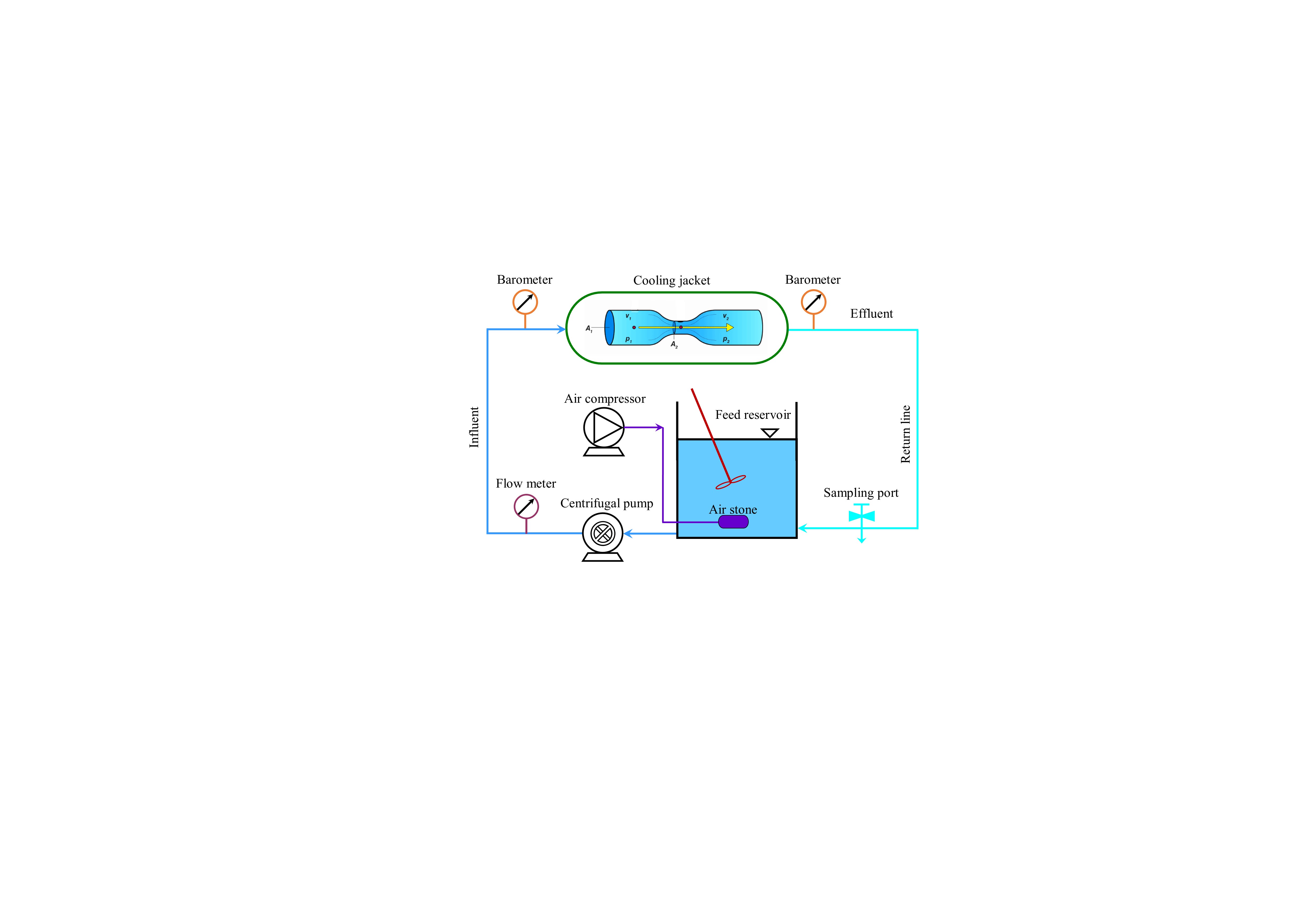


Fig. S2. Schematic diagram of the HC system.





Fig. S3. First-order kinetic curves of the degradation of DR83 under different solution pH. Experimental conditions: [DR83]_0_ = 20.0 mg/L, [ZVI]_0_ = 50.0 mg/L, [sulfite]_0_ = 50.0 mg/L, air flow = 1.5 L/min, solution pH_0_ = 3.0-9.0, and reaction time = 5-60 min


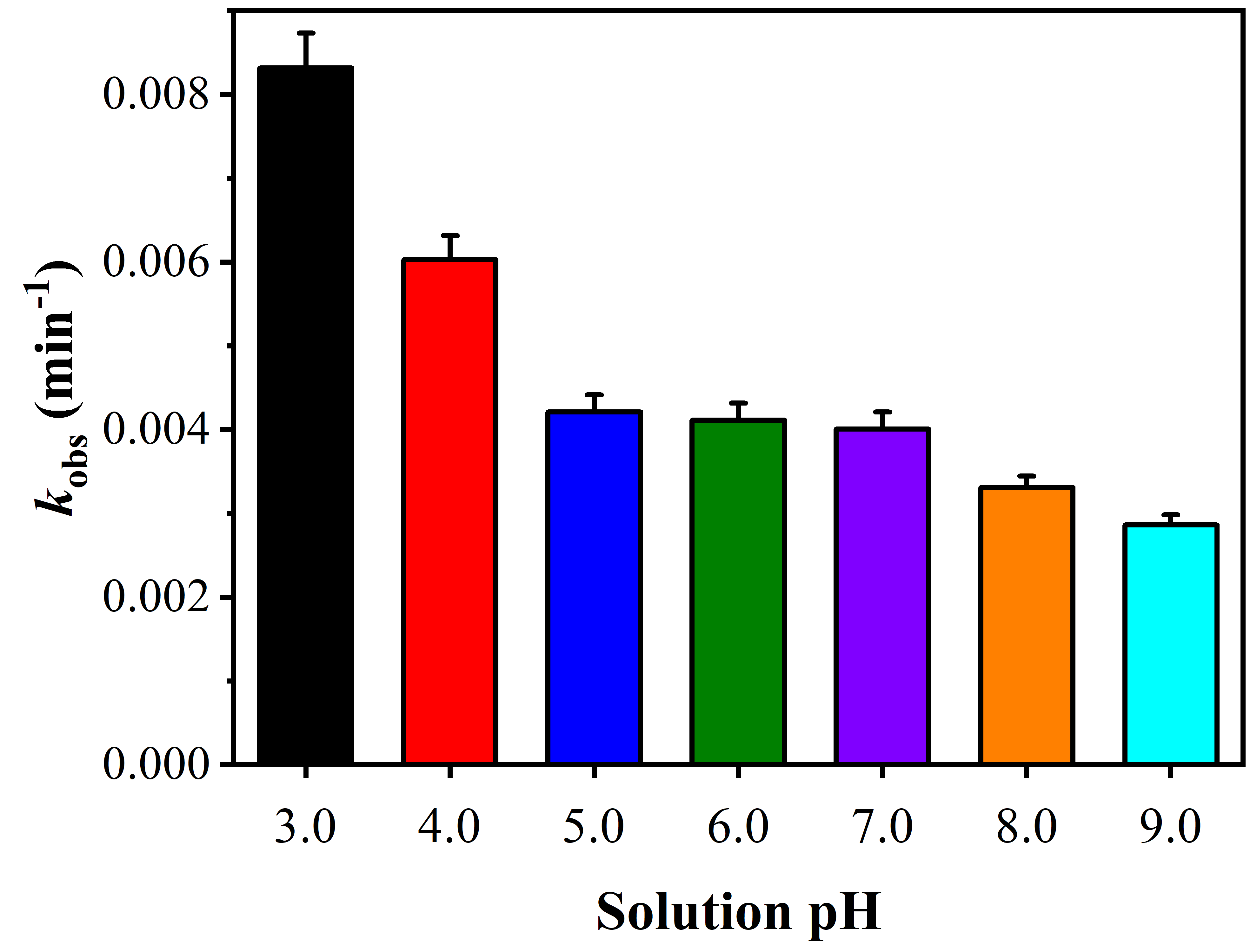


Fig. S4. Effect of solution pH on the kinetic rates of DR83 degradation. Experimental conditions: [DR83]_0_ = 20.0 mg/L, [ZVI]_0_ = 50.0 mg/L, [sulfite]_0_ = 50.0 mg/L, air flow = 1.5 L/min, solution pH_0_ = 3.0-9.0, and reaction time = 5-60 min





Fig. S5. First-order kinetic curves of the degradation of DR83 under different doses of ZVI. Experimental conditions: [DR83]_0_ = 20.0 mg/L, [ZVI]_0_ = 50.0-300.0 mg/L, [sulfite]_0_ = 50.0 mg/L, air flow = 1.5 L/min, solution pH = 3.0, and reaction time = 5-60 min.





Fig. S6. First-order kinetic curves of the degradation of DR83 under different sulfite doses. Experimental conditions: [DR83]_0_ = 20.0 mg/L, [ZVI]_0_ = 200.0 mg/L, [sulfite]_0_ = 50.0-300.0 mg/L, air flow = 1.5 L/min, solution pH = 3.0, and reaction time = 5-60 min.





Fig. S7. First-order kinetic curves of the degradation of DR83 under different concentrations of DR83. Experimental conditions: [DR83]_0_ = 20.0-200.0 mg/L, [ZVI]_0_ = 200.0 mg/L, [sulfite]_0_ = 250.0 mg/L, air flow = 1.5 L/min, solution pH = 3.0, and reaction time = 5-60 min.


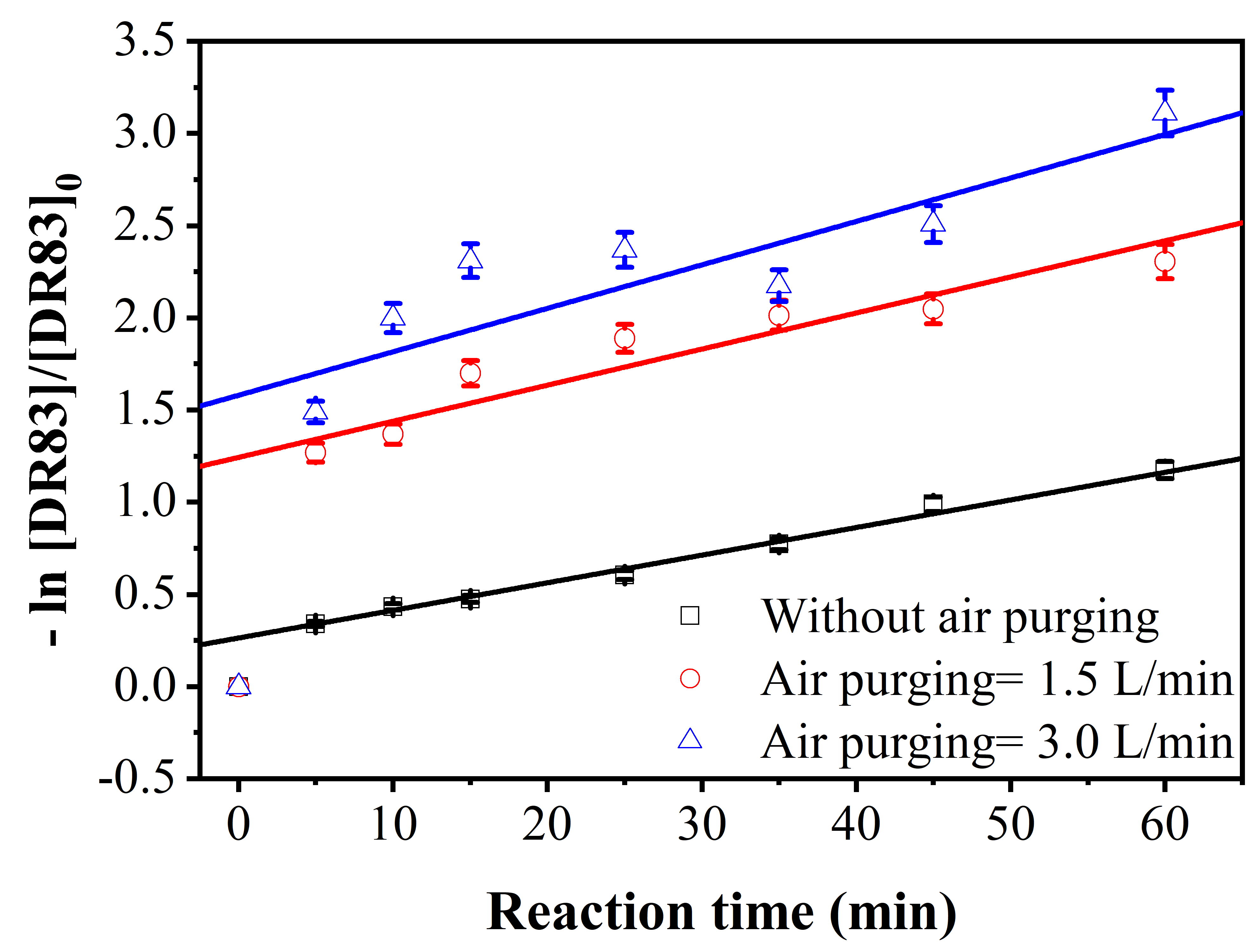


Fig. S8. First-order kinetic curves of the degradation of DR83 under different air purging. Experimental conditions: [DR83]_0_ = 50.0 mg/L, [ZVI]_0_ = 200.0 mg/L, [sulfite]_0_ = 250.0 mg/L, solution pH = 3.0. and reaction time = 5-60 min.


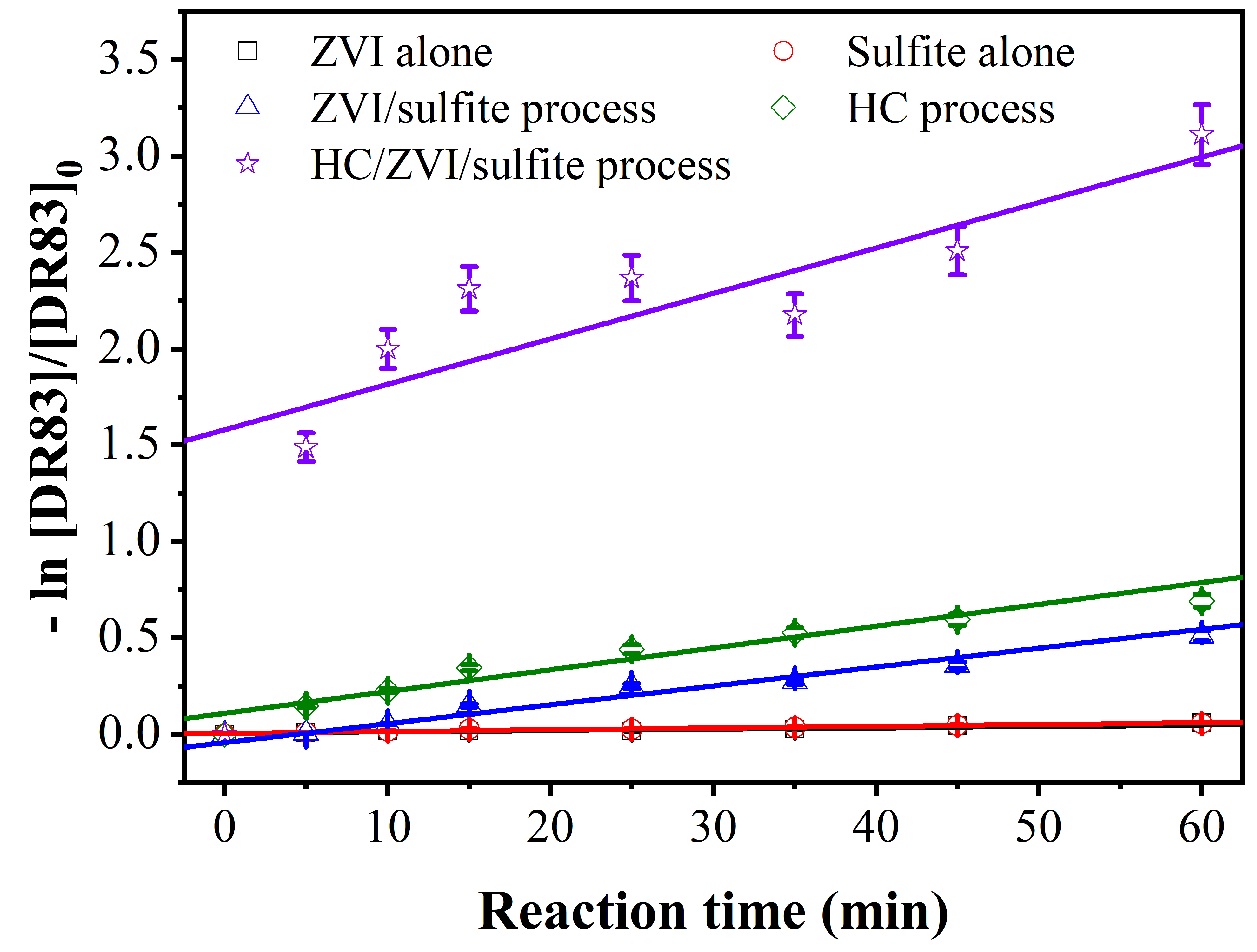


Fig. S9. First-order kinetic curves of the degradation of DR83 through different processes. Experimental conditions: [DR83]_0_ = 50.0 mg/L, [ZVI]_0_ = 200.0 mg/L, [sulfite]_0_ = 250.0 mg/L, pH = 3.0, air flow = 3.0 L/min, and reaction time = 5-60 min.


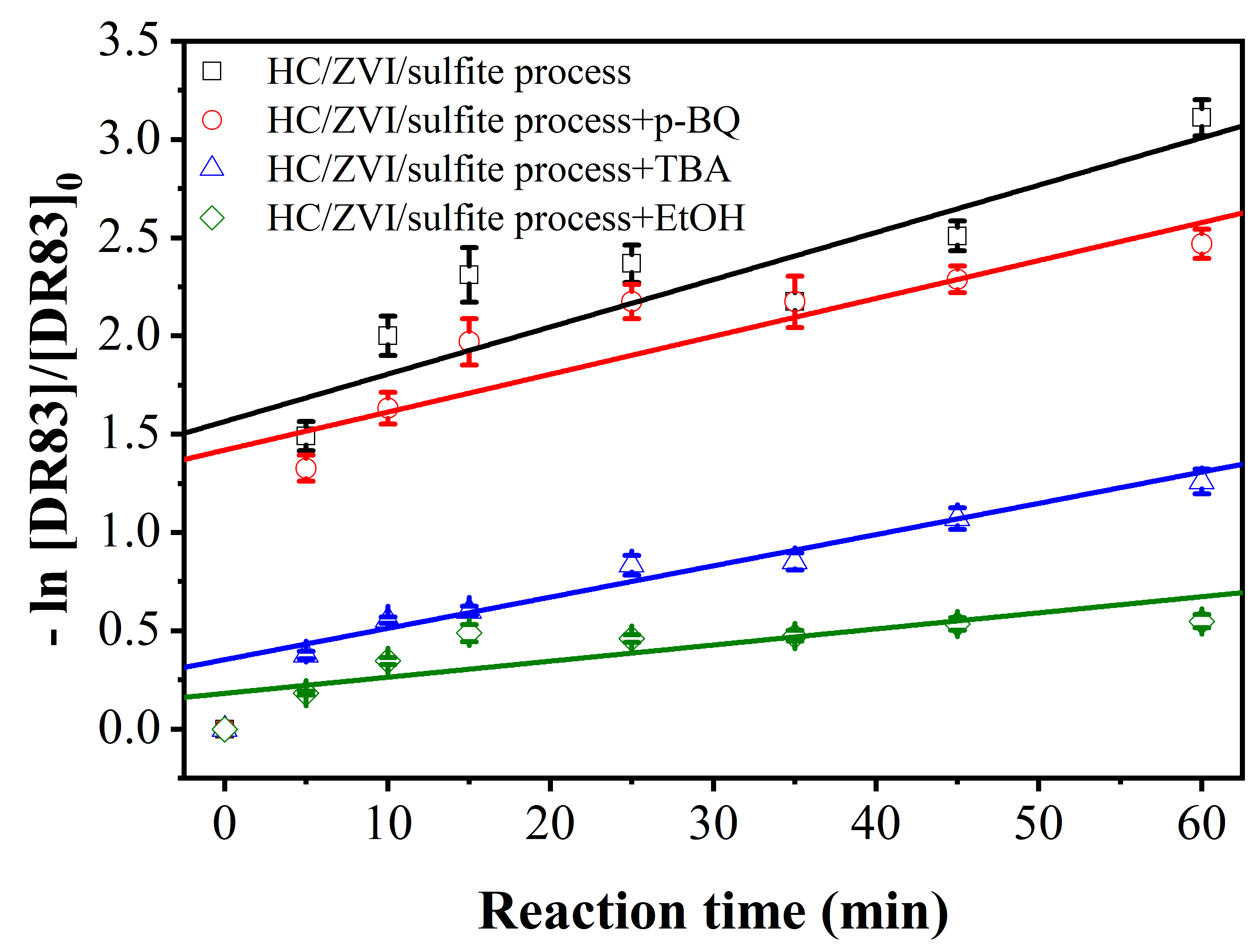


Fig. S10. First-order kinetic curves of the degradation of DR83 in the presence of radical scavengers. Experimental conditions: [DR83]_0_ = 50.0 mg/L, [ZVI]_0_ = 200.0 mg/L, [sulfite]_0_ = 250.0 mg/L, pH = 3.0, air flow = 3.0 L/min, concentration of scavengers = 15 mM and reaction time = 5-60 min.


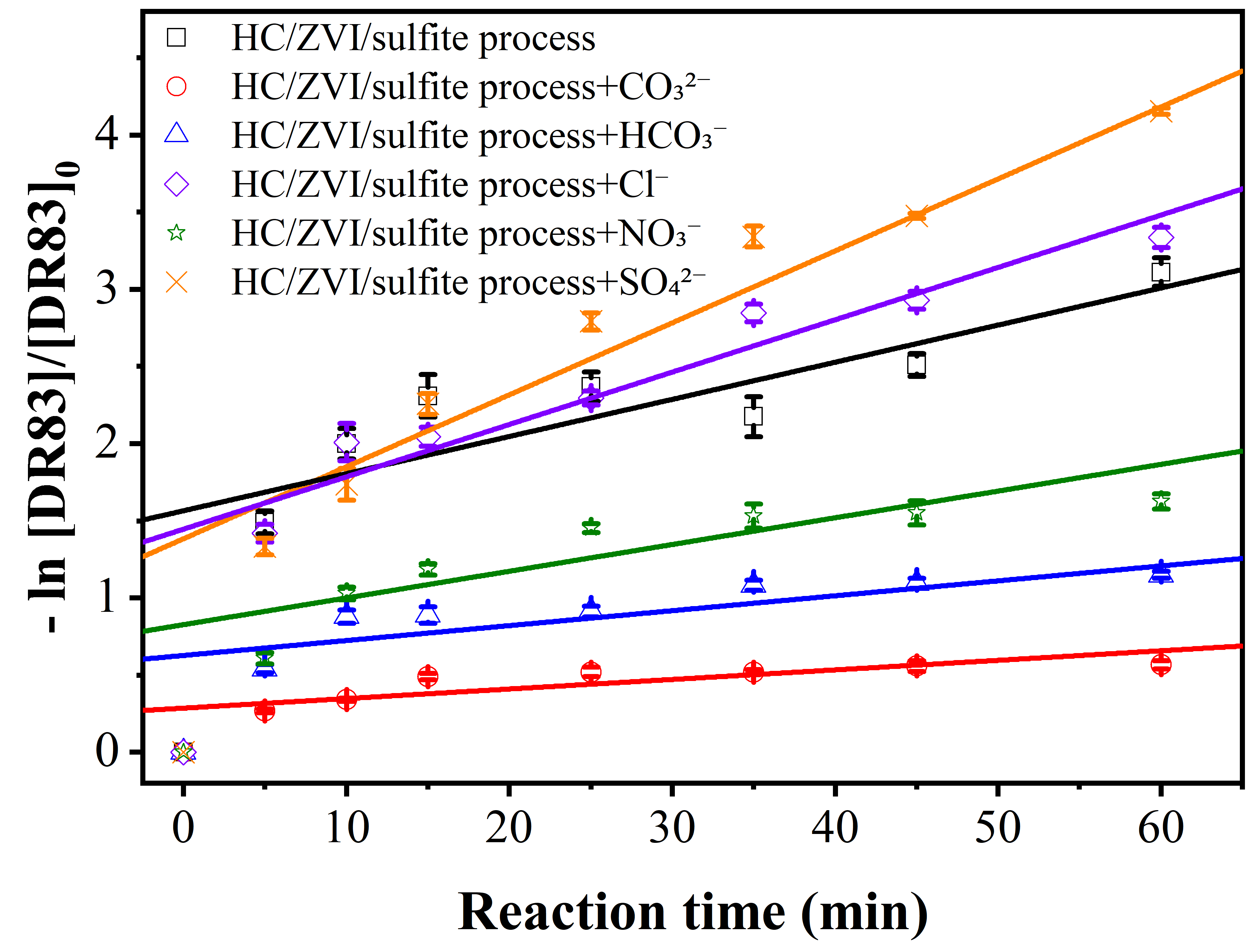


Fig. S11. Influence of coexisting anions on DR83 degradation. Experimental conditions: [DR83]_0_ = 50.0 mg/L, [ZVI]_0_ = 200.0 mg/L, [sulfite]_0_ = 250.0 mg/L, pH = 3.0, air flow = 3.0 L/min, concentration of anions = 15 mM, and reaction time = 5-60 min.
